# The plasticity of primary microglia and their multifaceted effects on endogenous neural stem cells in vitro and in vivo

**DOI:** 10.1186/s12974-018-1261-y

**Published:** 2018-08-13

**Authors:** Sabine Ulrike Vay, Lea Jessica Flitsch, Monika Rabenstein, Rebecca Rogall, Stefan Blaschke, Judith Kleinhaus, Noémie Reinert, Annika Bach, Gereon Rudolf Fink, Michael Schroeter, Maria Adele Rueger

**Affiliations:** 1 Department of Neurology, University Hospital of Cologne, Kerpener Str. 62, 50924 Cologne, Germany; 2 Cognitive Neuroscience, Institute of Neuroscience and Medicine (INM-3), Research Centre Juelich, Juelich, Germany

**Keywords:** Neuroinflammation, Neuroprotection, Stem cell-mediated regeneration, M1 microglia, M2 microglia, Hybrid microglia, Cerebral ischemia

## Abstract

**Background:**

Microglia—the resident immune cells of the brain—are activated after brain lesions, e.g., cerebral ischemia, and polarize towards a classic “M1” pro-inflammatory or an alternative “M2” anti-inflammatory phenotype following characteristic temporo-spatial patterns, contributing either to secondary tissue damage or to regenerative responses. They closely interact with endogenous neural stem cells (NSCs) residing in distinct niches of the adult brain. The current study aimed at elucidating the dynamics of microglia polarization and their differential effects on NSC function.

**Results:**

Primary rat microglia in vitro were polarized towards a M1 phenotype by LPS, or to a M2 phenotype by IL4, while simultaneous exposure to LPS plus IL4 resulted in a hybrid phenotype expressing both M1- and M2-characteristic markers. M2 microglia migrated less but exhibit higher phagocytic activity than M1 microglia. Defined mediators switched microglia from one polarization state to the other, a process more effective when transforming M2 microglia towards M1 than vice versa. Polarized microglia had differential effects on the differentiation potential of NSCs in vitro and in vivo, with M1 microglia promoting astrocytogenesis, while M2 microglia supported neurogenesis. Regardless of their polarization, microglia inhibited NSC proliferation, increased NSC migration, and accelerated NSC differentiation.

**Conclusion:**

Overall, this study shed light on the complex conditions governing microglia polarization and the effects of differentially polarized microglia on critical functions of NSCs in vitro and in vivo. Refining the understanding of microglia activation and their modulatory effects on NSCs is likely to facilitate the development of innovative therapeutic concepts supporting the innate regenerative capacity of the brain.

**Electronic supplementary material:**

The online version of this article (10.1186/s12974-018-1261-y) contains supplementary material, which is available to authorized users.

## Background

Cerebral ischemia results not only in damage and neuronal loss but also in sustained neuroinflammation lasting for months after stroke [1–3]. In neurodegenerative disorders, ongoing inflammatory processes accompany and exacerbate neuronal loss [4–6]. On the one hand, this (sterile) neuroinflammation eliminates degenerating tissue, thus supporting a restorative milieu for neuroprotection, axonal regeneration, remyelination, and stem cell-mediated tissue repair. On the other hand, these immune processes are often deregulated, potentially also leading to detrimental effects. Hence, immune responses have to be tightly regulated to adequately promote beneficial effects and to avoid secondary damage [7]. Within the central nervous system (CNS), microglia are the resident immune cells that mediate and regulate inflammatory processes. In a resting state, microglia are involved in the maintenance of cell homeostasis surveying their microenvironment and communicating with the neuronal tissue via extremely motile processes and protrusions [8]. Furthermore, microglia play a pivotal role in neuronal development, function, and synaptic plasticity in the developing as well as the mature healthy brain [9–12]. After brain injury, microglia are activated and exert either neurotoxic or neuroprotective effects, depending on their activation phenotype [13, 14]. In accordance with the nomenclature of peripheral macrophages, activated microglia that secrete pro-inflammatory cytokines and nitric oxide (NO) and lead to neuronal loss and to impairment of tissue repair are categorized as “classically” activated (M1). The “alternative” activation results in M2 microglia polarization, releasing neurotrophic factors and promoting healing and repair [13, 15]. The course of microglia polarization after, e.g., cerebral ischemia, follows distinct temporo-spatial patterns [2, 16, 17], but seemingly lacks a signal for termination of those inflammatory processes, resulting in additional tissue damage. Moreover, poor spontaneous recovery from cerebral injury is associated with the incorrect timing of microglia recruitment, excessive or insufficient numbers of microglia, and an inappropriate microglia polarization [18]. However, the dichotomy between those opposed microglia phenotypes potentially offers new therapeutic options to support regenerative processes: A better understanding of the distinct components of microglia activation and polarization might enable us to reduce harmful and to enhance beneficial functions. Though the characterization of the different microglia phenotypes M1 and M2 has been a matter of intense investigation over the last years [13, 19, 20], data are still scarce on the course and the reversibility of microglia polarization as well as on their capability of preserving molecular memories of previous stimuli [21].

In the intricate course of tissue regeneration, microglia play a key role due to their close interactions with endogenous neural stem cells (NSCs) mediating repair. Activated microglia release soluble factors mobilizing endogenous NSCs from their niches after stroke, namely from the subventricular zone (SVZ) adjacent to the lateral ventricles as well as from the subgranular zone (SGZ) of the hippocampal dentate gyrus [22]. Mobilized NSCs proliferate and differentiate into neurons, astrocytes and oligodendrocytes, thereby supporting regeneration [23–26]. Adding to this, according to our current understanding, NSCs do not only mediate their beneficial effects via neurogenesis and neuronal replacement, but rather exhibit immunomodulatory, neuroprotective, and re-myelinating properties [18, 27]. Further evidence suggests that NSCs regulate microglia function and activity [28]. In turn, the neuroinflammatory milieu created by activated microglia recruits endogenous NSCs to the lesion site and modulates their inherent capacity of regeneration. Accordingly, activated microglia and their secreted cytokines and soluble factors exert crucial influence on NSCs [18, 29]. We recently investigated the effects of pro-inflammatory effectors (IL1β, TNF-α, and IL6) on the differentiation potential of NSCs, revealing a significant inhibition of neurogenesis and an enhanced as well as accelerated astrocytogenesis, which was partly counteracted by minocycline [30]. However, depending on their activation type and surrounding milieu, microglia release different panels of soluble factors, suggesting more complex interactions with NSCs. Altogether, both microglia and NSCs are involved in regenerative processes after stroke while maintaining a multifaceted cross-talk. Under the hypothesis that NSCs are the key effector cells in brain regeneration, and that microglia as their key regulators ultimately determine their regenerative potential, the aim of our present study was to comprehensively investigate the effects of various microglia polarization phenotypes on primary NSCs in vitro and in vivo.

## Methods

### Microglia isolation and cultivation

Pure neonatal microglia cultures were obtained from the cortices of neonatal Wistar rats (P1-P3) as previously described in detail [31]. In brief, rat cortices were incubated in trypsin/EDTA solution (0.05% trypsin, 0.02% EDTA) for 15 min at 37 °C. Addition of the culture medium (Dulbecco’s essential medium (DMEM) with the addition of 10% fetal calf serum (FCS), 1% penicillin/streptomycin and 2 mM l-glutamine) stopped the reaction. The cortices were dissociated by repeated up- and down-pipetting with a glass pipette. The resulting cell suspension was centrifuged at 1200 rpm for 2 min. Cells were re-suspended in DMEM (10% FCS, 1% penicillin/streptomycin, 2 mM l-glutamine) and grown at 37 °C with 5% CO_2_ for 8–10 days, while the culture medium was changed every 3 days. This prolonged cultivation approach promoted a selective growth of astrocytes and microglia. To harvest pure microglia from this initial co-culture, culture flasks were shaken for 1 h at 250 rpm on an orbital shaker (37°) to detach microglia. The medium containing the layer of detached microglia was collected and immediately centrifuged for 2 min at 1200 rpm. The supernatant was removed, and the obtained pure microglia pellet was re-suspended in fresh culture medium and seeded into subcultures. Flasks were refilled with culture medium, and microglia harvesting was repeated for maximal three times at intervals of 3 days. All experiments were performed with the purified microglia cultures.

Twenty-four hours after seeding microglia in subculture (50,000 cells/well on a 24-well plate), cells were left unstimulated (control) or stimulated with 1 or 10 ng/ml lipopolysaccharide (LPS derived from *Escherichia coli* 0111: B4, Sigma Aldrich, St. Louis, USA) to polarize to M1, or with 50 ng/ml recombinant rat interleukin-4 (IL4; R&D Systems, Minneapolis, USA) to polarize to M2, or combined LPS plus IL4 (1 or 10 ng/ml LPS and 50 ng/ml IL4). For migration assays, cells were treated immediately at seeding. For pre- and post-stimulation experiments of M1 microglia with recombinant rat IL4 (50 ng/ml), the cells were initially stimulated 2 h after seeding microglia subcultures, before a second stimulation was performed after another 24 h. At the same time of the second stimulation, the culture medium was routinely changed. Conducting experiments on acute or chronic inflammatory stimuli, the culture medium was changed every 24 h with or without LPS treatment (10 ng/ml) or recombinant rat IL4 treatment (50 ng/ml). Experiments were generally stopped 24 h after the last stimulation.

To obtain “conditioned medium” for further experiments in vitro and in vivo, the microglia were stimulated in serum-free DMEM/F12 medium plus 1% N2 supplement, 1% penicillin/streptomycin, 0.6 mM l-glutamine, and 1% sodium pyruvate (Thermo Fisher Scientific, Waltham, USA). Twenty-four hours after initial stimulation, the conditioned medium—containing the entire microglia secretome—was removed and centrifuged at 11,900*g*, and the supernatant was stored at − 80 °C.

### Neural stem cell (NSC) isolation and cultivation

NSCs from fetal Wistar rat cortices were derived from embryonic day 13.5 as described previously [32]. Briefly, fetal cortices were dissociated in DMEM/F12 medium plus 1% N2 supplement, 1% penicillin/streptomycin, 0.6 mM l-glutamine, and 1% sodium pyruvate (Thermo Fisher Scientific, Waltham, USA) by repeated up- and down-pipetting and the resulting cell suspension was sown on 10-cm Petri dishes for expansion. Human recombinant basic fibroblast growth factor (FGF2; 10 ng/ml, Thermo Fisher Scientific, Waltham, USA) was added throughout culturing, and the medium was changed every other day. For all experiments, NSCs of the second to fourth passage were used.

#### Monolayer culture

For monolayer cultures, dishes or wells were pre-coated with l-poly-ornithine (15%, Sigma Aldrich, St. Louis, USA) and bovine fibronectin (2.5 mmol/l, R&D Systems, Minneapolis, Canada). NSCs were grown at 37 °C with 5% CO2. For differentiation, proliferation, and survival experiments, cells from the second or third passage were sown on pre-coated chamber slides or 24-well plates. After 24 h, NSCs were stimulated with conditioned medium of unstimulated microglia (conditioned medium [untreated]), LPS-stimulated microglia (conditioned medium [LPS]), IL4-stimulated microglia (conditioned medium [IL4]), or microglia stimulated with LPS and IL4 (conditioned medium [LPS + IL4]). NSCs stimulated with sheer 10 ng/ml LPS or 50 ng/ml IL4 as well as unstimulated NSCs served as controls. For analyzing migration potential and neurosphere growing, cells were immediately stimulated when seeded.

#### Neurosphere culture

To assess the quality of neurosphere growth, 50,000 NSCs/well were cultured in a non-coated 24-well plate. In the presence of FGF2, NSCs were treated as described above (LPS alone, IL4 alone, or conditioned medium from microglia pre-treated with LPS and/or IL4). After 48 h, qualitative digital images were taken with a Keyence BZ-9000 inverted fluorescence microscope (Keyence Osaka, Japan).

#### NSC differentiation assay

NSCs were re-plated in chamber slides in the presence of FGF2 (15,000 cells/chamber). After 24 h, differentiation was initiated by withdrawal of the mitogen FGF2, and NSCs were stimulated as described above (LPS alone, IL4 alone, or conditioned medium from microglia pre-treated with LPS and/or IL4) for 24 h. After 5 days of differentiation, NSCs were fixed with paraformaldehyde (4%, Electron Microscopy Sciences, Hatfield, USA) and immunocytochemically stained to visualize NSCs, young neurons, oligodendrocytes, and astrocytes (see immunocytochemistry).

### Griess assay

NO release from microglia was quantified by photometrical detection of NO using a Griess reagent kit (Biotium, Hayward, USA). Twenty-four hours after the last stimulation of microglia, the supernatant was collected, and the content of NO was measured in accordance with the manufacturer’s protocol. The optical density (OD) of each sample was measured at 548 nm in a plate reader (FLUOstar Omega, BMG LABTECH, Ortenberg, Germany). Mean values ± standard error of the mean (SEM) were established among equally treated samples. Each experiment was conducted in triplicate.

### Enzyme-linked immunosorbent assays (ELISA)

Pro-inflammatory cytokines (tumor necrosis factor-α (TNF-α) and interleukin 6 (IL-6)) were measured in the culture medium of stimulated and unstimulated microglia 24 h after the last stimulation using the rat TNF-α DuoSet ELISA Development System and the IL-6 DuoSet ELISA Development System (Cat# DY510 and DY506, R&D Systems, Minneapolis, MN). Insulin-like growth factor 1 (IGF1) was measured as an anti-inflammatory product using the mouse/rat IGF1 Quantikine ELISA Kit (Cat# MG100, R&D systems, Minneapolis, Canada). Experiments were conducted according to the manufacturer’s protocol. The OD of each sample was measured using the plate reader, and cytokine concentrations of the samples were calculated on the basis of standard curves. Mean values ± SEM were established among equally treated samples. Each experiment was conducted in triplicate.

### Phagocytosis assay

Phagocytic activity of untreated and treated microglia was assessed using a phagocytosis assay (CytoSelect™ 96-well Phagocytosis Assay, Cell Biolabs, San Diego, CA, USA) according to the manufacturer’s protocol. Briefly, phagocytic activity was measured by the amount of engulfed pre-labeled zymosan substrate uptake after 2 h of incubation by colorimetrical detection after blocking external zymosan particles. The OD of each sample was measured at 405 nm using the plate reader. The resulting mean values ± SEM were established among equally treated samples and compared to microglia not treated with zymosan.

### Real-time quantitative PCR (RT-qPCR)

RNA from cultivated cells was isolated 24 h after the last treatment (LPS, IL4, LPS + IL4) using the GeneUP total RNA mini Kit (Biotechrabbit, Henningsdorf, Germany), following the manufacturer’s protocol. Total RNA concentration and purity were evaluated photometrically. Total RNA (10 ng) was converted to c-DNA by reverse transcription with the QuantiTect reverse transcription Kit (Qiagen Hilden, Germany) in accordance with the manufacturer’s recommendations. All primers used in this study were obtained from Biolegio (Nijmegen, The Netherlands). Primer sequences and PCR parameters are enlisted in Table 1. The samples were amplified and quantified on a Rotorgene 2000 cycler (Corbett, Sydney, Australia). PCR product integrity was evaluated by melting point analysis and agarose gel electrophoresis. The threshold cycle (CT) was normalized to ribosomal protein L13a (RPL13a; ΔCT) and the experimental control condition (ΔΔCT). Mean fold changes are depicted as 2^(−ΔΔCT)^. RT-qPCR was performed in technical triplets, and each experiment was conducted in biological triplicate. Mean values ± SEM were calculated for all samples.

**Table 1:** Used primers and parameters of RT-qPCR

RNA	Sequences forward/backward 5′-3′	Temperature (°C) step 1/2/3	Duration (s) step 1/2/3	Accession number
iNOS	GCTTGTCTCTGGGTCCTCTG/CTCACTGGGACAGCACAGAA	95/59/72	15/15/45	NM_012611.3
CD206	AACAAGAATGGTGGGCAGTC/CCTTTCAGTCCTTTGCAAGC	95/56/72	15/15/45	NM_001106123.2
Ki67	TCTTGGCACTCACAGTCCAG/ GCTGGAAGCAAGTGAAGTCC	95/58/72	15/15/45	NM_001271366.1
RPL13a	TCTCCGAAAGCGGATGAACAC/CAACACCTTGAGGCGTTCCA			NM_173340.2

### Live/dead assay

Cells were sown on 24-well plates (20,000 NSCs/well, 50,000 microglia cells/well). To assess the toxic effects of stimulatory agents (LPS, IL4, LPS + IL4, or conditioned medium), dead cells were stained with propidium iodide (Life Technologies, Darmstadt, Germany) 24 h after treatment. All cells, irrespective of viability, were counterstained with Hoechst 33342 (Life Technologies, Darmstadt, Germany). Representative pictures were taken using the inverted fluorescence phase-contrast microscope. Ten images per well were taken, and both Hoechst-stained as well as propidium iodide-stained cells were counted manually. The ratio of propidium iodide positive on total cell count provided the proportion of cell death. The experiment was performed in triplicate with 4 wells per condition. The resulting mean values ± SEM were established among equally treated samples.

### Lactate dehydrogenase (LDH) assay

In addition to the live/dead assay, cell death (NSCs or microglia) was indirectly assessed by measuring LDH release to the media using a colorimetric assay (Pierce LDH assay kit, Thermo Scientific, Waltham, USA) 24 h after treatment with LPS, IL4, LPS + IL4, or conditioned medium. The experiment was performed according to the manufacturer’s protocol. The intensity of the red color formed in the assay was measured at a wavelength of 490 nm (FLUOstar Omega, BMG LABTECH, Ortenberg, Germany), being proportional to LDH activity and thus correlating with the number of damaged cells. The experiment was performed in triplicate with 4 wells per condition. The resulting mean values ± SEM were established among equally treated samples.

### Bromodeoxyuridine (BrdU) proliferation assay

BrdU (Sigma Aldrich, St. Louis, USA) served to label and quantify proliferating microglia or NSCs. Cells were sown on 24-well plates with glass coverslips inside the wells (20,000 NSCs/well, 50,000 microglia cells/well). Eighteen hours after treatment (LPS, IL4, LPS + IL4, or conditioned medium), 10 mM BrdU was added to each well. After 6 h of incubation, the experiment was stopped by cell fixation with 4% PFA and cells were stained for incorporated BrdU (see below). The ratio of BrdU-positive on total cell count provided the proportion of proliferating cells. The experiment was performed in triplicate with 4 wells per condition. The resulting mean values ± SEM were established among equally treated samples.

### Immunocytochemistry

The purity of the NSC and microglia cultures was regularly verified as described previously [30, 31]. Microglia cells were stained for ionized calcium-binding adapter molecule 1 (Iba1; rabbit polyclonal antibody, dilution 1:1000, Cat#. 019-19741, RRID:AB_839504, WAKO, Osaka, Japan) and inducible nitric oxide synthetase (iNOS, rabbit monoclonal antibody, dilution 1:1000, Cat# 178945, Abcam, Milton, UK). To assess the proliferation rate via bromodeoxyuridine (BrdU) incorporation, microglia or NSCs were stained with anti-BrdU (mouse monoclonal, clone BU-33, dilution 1:200, Cat# B8434, RRID:AB_476811, Sigma Aldrich, St. Louis, USA). To differentiate the cell types after NSC differentiation, cells were stained for markers to identify young neurons (neuron-specific beta-III tubulin 1 monoclonal antibody (mAb); mouse anti-TuJ1; dilution 1:100, Cat# MAB1195, RRID:AB_357520, R&D Systems, Minneapolis, USA), astrocytes (rabbit anti-glial fibrillary acidic protein (GFAP), clone GA5; dilution 1:1000, Cat# MAB360, RRID:AB_11212597, Millipore, Billerica, USA), oligodendrocytes (mouse anti-2′,3′-cyclic-nucleotide 3′-phosphodiesterase (CNPase); clone 11-5B, dilution 1:500, Cat# MAB326, RRID:AB_2082608, Millipore, Billerica, USA), and undifferentiated NSCs (mouse anti-sex determining region Y-box 2 (SOX2) mAb, dilution 1:100, Cat# MAB2018, RRID:AB_358009, R&D Systems, Minneapolis, USA). For visualization, fluorescein-labeled anti-mouse immunoglobulin G (IgG) or anti-rabbit IgG were used (dilution 1:200, Cat# A21124, RRID:AB_2535766 and Cat# A21135, RRID:AB_1500827, Thermo Fisher Scientific, Waltham, USA). All cells were counterstained with Hoechst 33342.

Ten pictures of each sample were taken using the inverted fluorescence phase-contrast microscope, and at least 250 total cells/sample and experiment were counted manually. To measure the dendritic length of stained neurons, the Image J software (RRID:SCR_003070, NIH, Bethesda, MD, USA) was used. All immunocytochemical experiments were performed in triplicate. The resulting mean values ± SEM were established among equally treated samples.

### Migration assay

The cell migration was analyzed via a trans-well migration assay using a modified Boyden chamber (CytoSelect™ 24-Well Cell Migration Assay, 8-μM pore size, Cell Biolabs, Inc., San Diego, USA), following the manufacturer’s protocol. Briefly, freshly collected cells were dissolved in serum-free culture medium within the inserted upper chamber (50,000 NSCs or 10,000 microglia). For microglia experiments, either 10 ng/ml LPS or 50 ng/ml recombinant rat IL4 were added to the upper chambers of the modified Boyden chamber. In NSC experiments, LPS 10 ng/ml, recombinant rat IL4 (50 ng/ml), or conditioned medium (of microglia pre-treated with LPS, IL4, or LPS + IL4) were filled into the lower chamber of the plate. Untreated cells served as a control, and 10% FBS-containing medium in the lower chamber served as a positive control. After 24 h, migrated cells on the lower side of the inserts were stained with crystal violet, extracted (extraction solution, Cell Biolabs, Inc., San Diego, USA), and quantified by photometrical detection (560 nm) in a plate reader (FLUOstar Omega, BMG LABTECH, Ortenberg, Germany). Mean values ± SEM were established among equally treated samples. Each experiment was conducted in triplicate.

### Animal experiments

Male adult Wistar rats weighing 280–310 g received intracerebroventricular (i.c.v.) injections of 3-μl conditioned medium of microglia pre-treated with LPS (*n* = 5) or conditioned medium of microglia pre-treated with IL4 (*n* = 5). One animal treated with conditioned medium of microglia pre-treated with LPS died due to unrelated causes during the course of the experiment. Animals receiving pure serum-free culture medium (DMEM/F12 medium plus 1% N2 supplement, 1% penicillin/streptomycin, 0.6 mM l-glutamine and 1% sodium pyruvate) served as control (*n* = 5). Over 5 days, all animals received daily intraperitoneal (i.p.) injections of 50 mg/kg/day bromodeoxyuridine (BrdU, Sigma Aldrich, St. Louis, USA) to label dividing cells. Rats were sacrificed for histology 8 days after i.c.v. injection.

### Immunohistochemistry

The procedures of the histological and immunohistological preparation were described previously in detail [30]. Briefly, rats were decapitated under deep anesthesia. The brains were rapidly removed, frozen in 2-methylbutane, and stored at − 80 °C until further processing. Coronal brain sections with a slice thickness of 10 μm were cut at 500-μm intervals, and the sections were stained with polyclonal antibody against doublecortin to identify neuroblasts (anti-DCX, dilution 1:500, Cat# ab18723, RRID:AB_732011, Abcam, Milton, UK) and with monoclonal antibody against BrdU (anti-BrdU, clone BU1/75, dilution 1:500, Cat# ab6326, RRID:AB_305426, Abcam, Milton, UK) to assess proliferating cells. Furthermore, sections were stained against GFAP to identify astrocytes (anti-GFAP, dilution 1:500, Cat# Z0334, RRID:AB_10013382, Dako, Santa Clara, USA). For fluorescent visualization, fluorescein-labeled anti-mouse immunoglobulin G (IgG), anti-rabbit IgG, or anti-rat IgG were used (dilution 1:500, Cat# A21124, RRID:AB_2535766, Cat# A21135, RRID:AB_1500827, Cat# A11006, RRID:AB_2534074, Thermo Fisher Scientific, Waltham, USA). All cells, except of BrdU-stained slices were counterstained with Hoechst 33342. For visualization of anti-DCX, the ABC Elite kit (Vector Laboratories, Burlingame, USA) with diaminobenzidine (Sigma-Aldrich, Munich, Germany) as the final reaction product was used. The area containing doublecortin-positive cells in the SVZ was measured on a fixed length of 100 μm (μm^2^). Images of the SVZ were taken using the × 20 and × 40 objective, and DCX- and BrdU-positive cells were counted in the SVZ depicted in a 100 × 100 μm^2^ (= ROI, compare Fig. 7). GFAP-positive cells were counted in the SVZ as well as in the striatum (images taken with × 40 objective = ROI, compare Fig. 7).

### Data processing and statistical analyses

Raw numerical data and graphics were processed with Microsoft Excel (Version 2016, Microsoft Corp., Seattle, WA, USA). Images were edited with Adobe Photoshop (Version 7.0, RRID:SCR_014199). Microsoft PowerPoint (Version 2016, Microsoft Corp.) and PDF creator (PDF24, pdfforge GmbH, Hamburg, Germany) were used to provide figures.

Statistical analyses were performed with IBM SPSS Statistics (Version 24, International Business Machines Corp. IBM, RRID:SCR_002865, Armonk, USA). To determine whether variables met the assumptions of linear models, Kolmogorov-Smirnov or Shapiro-Wilk test for normal distribution and Levene’s test of variance homogeneity were carried out. If all variables analyzed met the assumption of normality, one-way analysis of variance (ANOVA) was conducted to compare multiple groups. ANOVA was followed up by pairwise comparisons using Tukey-honest significant difference or Game’s Howell test, which was used for variables with unequal variances. In case the parameters turned out to be not normally distributed, the non-parametric Kruskal-Wallis test was calculated and followed up by multiple comparisons. Statistical significance was set at less than the 5% level (*p* < 0.05).

## Results

### Activation of primary rat microglia in vitro: characterization of different polarization phenotypes

**Fig. 1:**
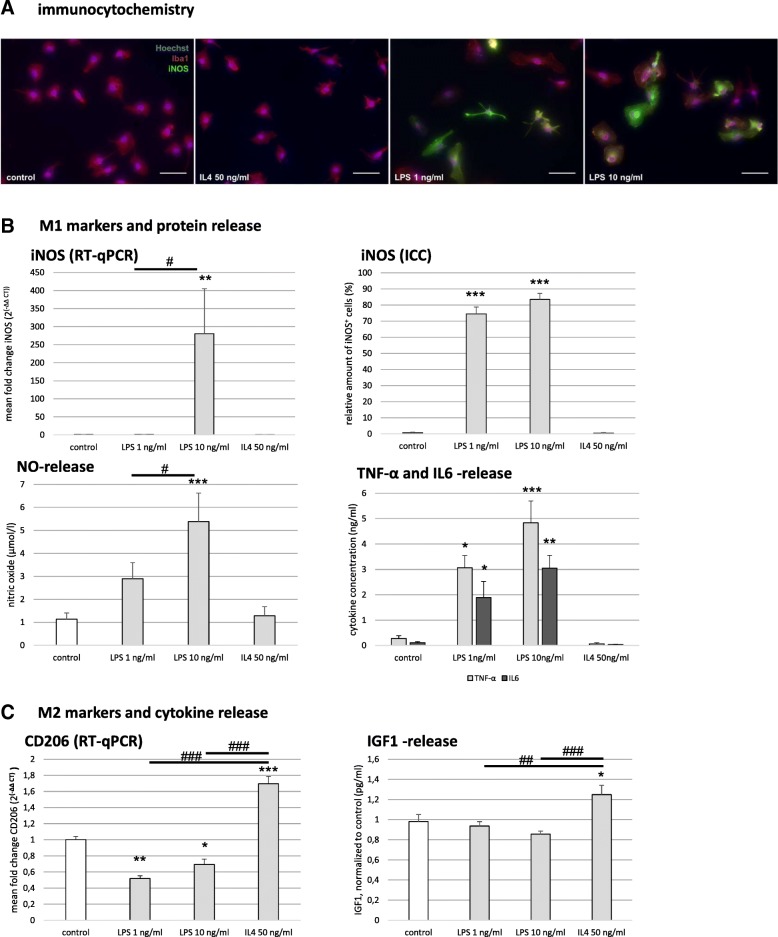
Polarization of primary microglia by lipopolysaccharide (LPS) and interleukin-4 (IL4). *p < 0.05, **p < 0.01, *** *p* < 0.001 compared to control; ^*#*^*p* < 0.05, ^##^*p* < 0.01, ^###^*p* < 0.001 compared to different experimental group as marked by horizontal bar*.* **a** Representative immunocytochemical stainings for the microglia marker “ionized calcium-binding adapter molecule 1” (Iba1; red), co-stained for “inducible nitric oxide (NO) synthetase” (iNOS; green), and Hoechst 33342 (Hoechst) as a nuclear counterstain (blue); scale bar = 50 μm. **b** Characterization of the M1 microglia phenotype by expression of iNOS, release of NO, and M1-characteristic cytokines after treatment with LPS (1 and 10 ng/ml) or IL4 (50 ng/ml). INOS expression was measured on the RNA level by real-time quantitative PCR (RT-qPCR; *n* = 3, *H*(3) = 25.828, *p* < 0.001) and on the protein level by immunocytochemistry (*n* = 3, *H*(3) = 97.262, *p* < 0.001). Release of NO was measured by Griess assay (μmol/l; *n* = 4, *H*(3) = 23.176, *p* < 0.001). Release of tumor necrosis factor-α (TNF-α; ng/ml, *n* = 3, *H*(3) = 22.112, *p* < 0.001) and interleukin-6 (IL6; ng/ml, *n* = 3, H(3) = 21.588, *p* < 0.001) were measured by enzyme-linked immunosorbent assay (ELISA). **c** Characterization of the M2 microglia phenotype by expression of CD206 and release of M2-characteristic cytokines after treatment with LPS or IL4. Regulation of CD206 expression on the RNA level was measured by RT-qPCR (representative experiment, *F* (3, 11) = 69.671, *p* < 0.001, *ω* = 0.972). Insulin-like growth factor 1 (IGF1) release was measured by ELISA (pg/ml, *n* = 3, *F* (3, 27) = 7.082, *p* < 0.001, *ω* = 0.63)

The purity of our primary microglia culture was assessed by immunocytochemical staining for Iba1, being expressed by > 99% of all control cells (Fig. 1a, left panel). Our first aim was to determine whether external stimuli could polarize microglia under certain culture conditions. To obtain a pro-inflammatory phenotype, we treated microglia with LPS at 1 or 10 ng/ml, respectively, to activate the Toll-like-receptor 4 (TLR4) expressed on microglia in rodent CNS [33]. To direct microglia towards an anti-inflammatory state, we stimulated the cells with 50 ng/ml IL4 as a characteristic anti-inflammatory cytokine. Any toxic effect of LPS or IL4 on microglia was excluded by LDH assay as well as by live/dead assay, yielding > 88% of live cells in all conditions after 24 h (n.s., Additional file 1: Figure S1A, B). To characterize the pro-inflammatory M1 microglia phenotype, we conducted RT-qPCR as well as immunocytochemical stainings for iNOS. Furthermore, we investigated the release of NO by Griess assay and release of TNF-α and IL6 by ELISA. To characterize the anti-inflammatory M2 phenotype, we assessed CD206 marker expression by RT-qPCR and IGF1 release by ELISA. LPS at the high dose of 10 ng/ml massively upregulated iNOS expression on both RNA and protein level (*p* < 0.01 and *p* < 0.001 respectively; Fig. 1a, b). Interestingly, lower doses of LPS (1 ng/ml) primarily upregulated iNOS protein expression (*p* < 0.001; Fig. 1a, b). Absolute NO concentration, TNF-α, and IL6 levels were boosted by LPS treatment in a dose-dependent manner (Fig. 1b). Noteworthy, untreated or IL4-treated microglia neither expressed iNOS, nor produced NO (Fig. 1a, b). On the contrary, microglia treated with LPS downregulated CD206 expression to ~ 50% (*p* < 0.01), and IGF1 release to ~ 80% in comparison to untreated control microglia (n.s., Fig. 1c), suggesting that untreated microglia constitutively expressed CD206 and released IGF1. IL4 treatment upregulated baseline expression of CD206 by ~ 1.7 fold (*p* < 0.001), and the release of IGF1 by ~ 1.2 fold (*p* < 0.05), compared to untreated control cells (Fig. 1c). These expression patterns suggest that microglia adopted a pro-inflammatory (M1) and an anti-inflammatory (M2) polarization phenotype by treatment with LPS and IL4, respectively.

**Fig. 2:**
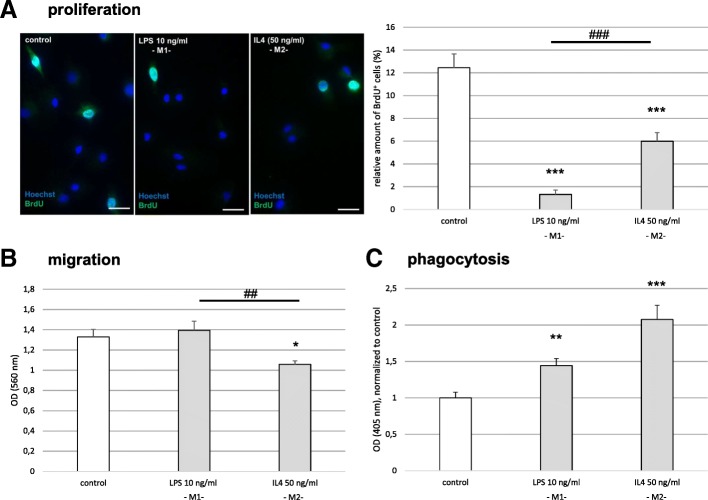
Functional changes in polarized microglia. * *p* < 0.05, **p < 0.01, *** *p* < 0.001 compared to control; ^*##*^*p* < 0.01, ^###^*p* < 0.001 compared to different experimental group as marked by horizontal bar. *OD* optical density*.* **a** Left panel: representative immunocytochemical images of bromodeoxyuridine (BrdU) incorporation: BrdU (green) identifies proliferating cells, Hoechst for nuclear counterstain (blue); scale bar = 50 μm. Right panel: proliferation rate of microglia after treatment with LPS (10 ng/ml) or IL4 (50 ng/ml) as measured by BrdU incorporation (*n* = 3; *H*(2) = 66.774, *p* < 0.001). **b** Migration of microglia in the Boyden chamber assay (*n* = 4, *F* (2, 33) = 6.244, *p* < 0.01, *ω* = 0.475). Data were blank corrected. **c** Phagocytic activity of microglia (*n* = 5, *H*(2) = 24.303, *p* < 0.001). Data were blank corrected and normalized to control

Next, we investigated the functional properties of untreated and polarized microglia. We used the higher dose of LPS (10 ng/ml) to obtain M1 microglia for those functional experiments. The proliferation rate of untreated microglia was on average 12% (during the 6 h allowed for BrdU incorporation). The proliferation rate of both M1 and M2 microglia was significantly reduced compared to the corresponding untreated control cells (< 2% for M1 and 6% for M2 [both *p* < 0.001], Fig. 2a). In the Boyden chamber assay, less M2 than M1 microglia migrated towards the lower chamber (*p* < 0.01, Fig. 2b). Both polarization phenotypes increased their phagocytic activity compared to the untreated control microglia, M2 microglia to an even higher extent than M1 microglia (1.4-fold for M1 [*p* < 0.01] and 2.1-fold for M2 [*p* < 0.001], compared to the untreated control cells, Fig. 2c).

### Microglia polarization is dynamic

**Fig. 3:**
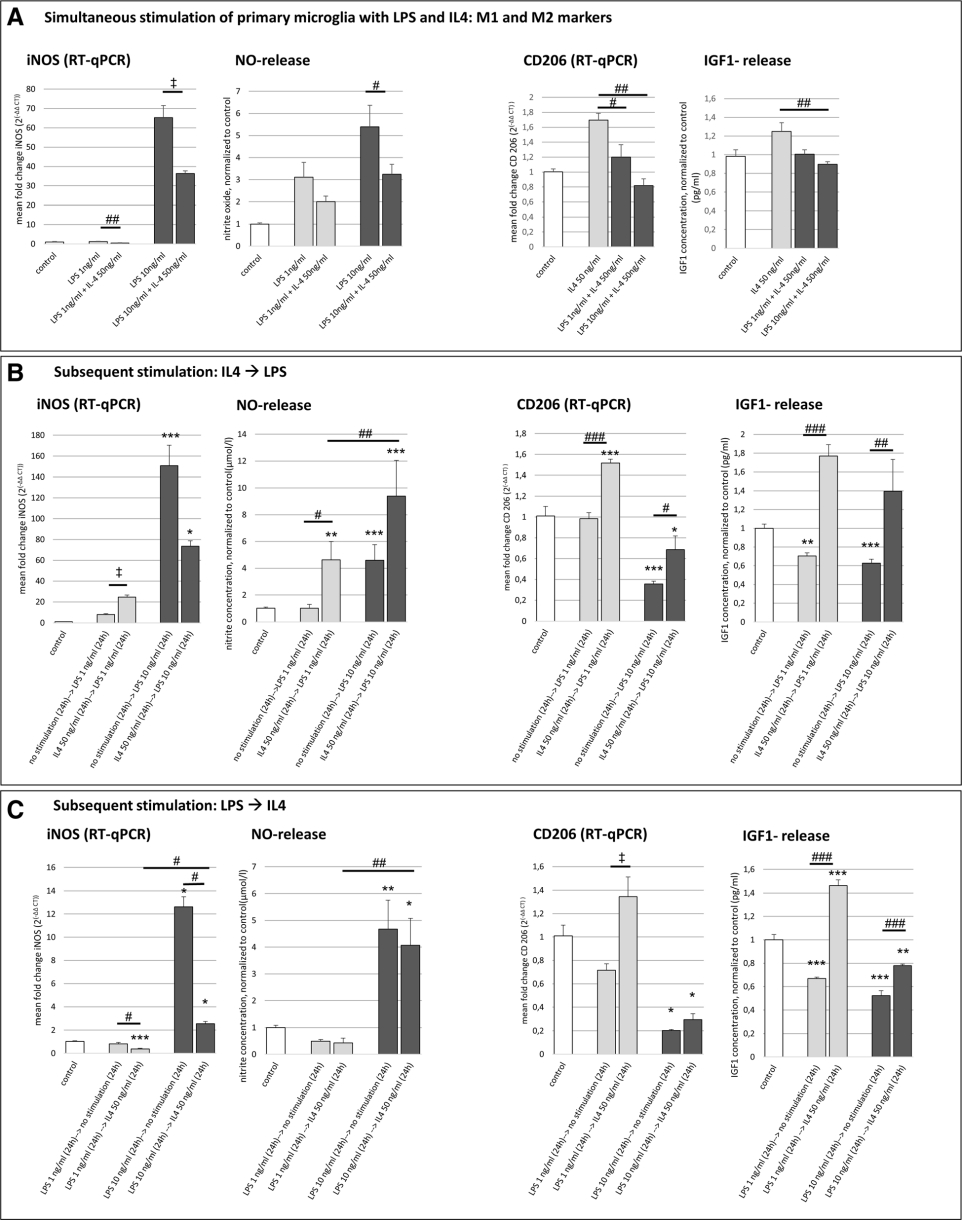
Switch between microglia phenotypes. *p < 0.05, **p < 0.01, ***p < 0.001 compared to control; ^*#*^*p* < 0.05, ^##^*p* < 0.01, ^###^*p* < 0.001 compared to different experimental group as marked by horizontal bar*;* ^‡^*p* < 0.05, ^‡‡^*p* < 0.01 between two groups (*t* test); only relevant significant values are highlighted. **a** Co-stimulation of microglia with LPS (1 or 10 ng/ml) plus IL4 (50 ng/ml) and resulting expression of M1 and M2 markers. Expression of iNOS (representative experiment, *F*(4, 14) = 99.481, *p* < 0.001, *ω* = 0.9815; *t* test: *t*(4) = 4.424, *p* < 0.05, *d* = − 2.62) and CD206 (representative experiment, *F*(3, 8) = 12.634, *p* < 0.01, *ω* = 0.86) were measured on the mRNA level by RT-qPCR. Release of NO was measured by Griess assay (μmol/l; *n* = 4, *H*(4) = 64.298, *p* < 0.001); IGF1 release was measured by ELISA (*n* = 3, *F*(3, 24) = 5.587, *p* < 0.01, ω = 0.57). **b** Stimulation with IL4 (50 ng/ml) and subsequently with LPS (1 or 10 ng/ml), and resulting expression of M1 and M2 markers. Expression of iNOS (representative experiment, *H*(4) = 13.500, *p* < 0.01; *t* test: *t*(4) = − 7.6, *p* < 0.01, *d* = 12.96) and CD206 (representative experiment, *F*(4, 10) = 49.487, *p* < 0.001, *ω* = 0.96) were measured on the mRNA level by RT-qPCR. NO release was measured by Griess assay (μmol/l; *n* = 7, *H*(4) = 28.615, *p* < 0.001); IGF1 release was measured by ELISA (*n* = 7, *H*(4) = 25.794, *p* < 0.001). **c** Stimulation with LPS (1 or 10 ng/ml) and subsequently with IL4 (50 ng/ml), and resulting expression of M1 and M2 markers. Expression of iNOS (*n* = 4, *F*(4, 27) = 400.684; *p* < 0.001, *ω* = 0.99) and CD206 (representative experiment, *F*(4, 10) = 27.475, *p* < 0.001, *ω* = 0.77; *t* test: *t*(4) = 3.56, *p* < 0.05, *d* = − 2.65) were measured on the mRNA level by RT-qPCR. NO release was measured by Griess assay (μmol/l; *n* = 6, *H*(4) = 27.456, *p* < 0.001); IGF1 release was measured by ELISA (pg/ml, *n* = 7, *F*(4, 35) = 121.074, *p* < 0.001, ω = 0.96)

To further explore the dynamics of microglia polarization, we simultaneously exposed microglia to LPS *plus* IL4. Simultaneous treatment with LPS plus IL4 led to a significant decrease of the M1-marker iNOS and, correspondingly less release of NO, TNF-α, and IL6, as compared to LPS-only exposed microglia (Fig. 3a, Additional file 2: Figure S2). This ability of IL4 to counteract the effects of LPS was observed for both the lower (1 ng/ml) and higher (10 ng/ml) LPS concentration. Similarly, simultaneous treatment with LPS plus IL4 led to a decrease of the M2-marker CD206 and reduced release of IGF1, as compared to IL4-only exposed microglia (Fig. 3a). This effect of LPS on M2-microglia was dose dependent, with application of 10 ng/ml LPS decreasing CD206 and IGF1 even further than the baseline levels of the untreated control cells, albeit only by trend (Fig. 3a).

As a next step, we investigated whether microglia that had been polarized by LPS or IL4 before as a “pre-stimulation” remained capable of reversing their polarization phenotype by subsequent stimulation with the respective other contra-acting agent (Fig. 3b, c). Microglia were exposed to LPS (1 or 10 ng/ml) or IL4 (50 ng/ml) for 24 h, followed by another 24 h of exposure to the respective other agent as an inverse trigger; untreated microglia served as control. Microglia exposed to 1 ng/ml LPS after pre-stimulation with IL4 expressed significantly higher levels of iNOS (~ 3-fold, *p* < 0.05) and NO (~ 4.5-fold, *p* < 0.05) than without pre-stimulation with IL4, surprisingly suggesting a sensitizing effect of IL4 pre-stimulation on M1 polarization (Fig. 3b). Interestingly, those microglia exposed to LPS after pre-stimulation with IL4 additionally still expressed the characteristic M2-markers CD206 and IGF1, in parallel to the M1-markers (Fig. 3b). On the other hand, microglia exposed to IL4 after pre-stimulation with 1 or 10 ng/ml LPS expressed significantly less iNOS (~ 2-fold and ~ 5-fold, respectively, both *p* < 0.05) and by trend less NO, compared to LPS treatment alone (Fig. 3c), suggesting that M1 polarization was at least in part reversed by IL4. In line with this, microglia exposed to IL4 after pre-treatment with LPS were still capable of upregulating M2-markers (Fig. 3c).

Another noteworthy aspect was observed in the different control groups of this experiment. One control group of microglia was treated with 10 ng/ml LPS for 24 h, then left in regular (control) media without stimulating agent for another 24 h (cf. Fig. 3c: “LPS 10 ng/ml ➔ no stimulation”). Another control group was grown in regular (control) media without stimulating agent for 24 h, then exposed to 10 ng/ml LPS for another 24 h (cf. Fig. 3b: “no stimulation ➔ LPS10 ng/ml”). When LPS was introduced first, then withdrawn later, iNOS was upregulated 12-fold compared to completely untreated controls (*p* < 0.05, Fig. 3c). However, when LPS was introduced only later to previously untreated cells, they upregulated iNOS by 150-fold (*p* < 0.001, Fig. 3b). Thus, data suggest that the timing of LPS exposure was critical for the amount of pro-inflammatory mediators produced by M1 microglia and that the polarization phenotype was not permanent, but dynamically changing over time.

### M1 polarization requires constant stimulation, while M2 polarization perpetuates itself

**Fig. 4:**
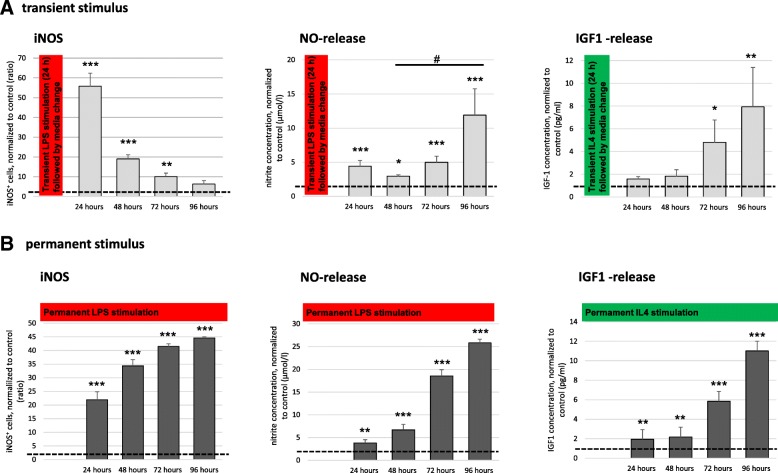
Microglia polarization kinetics. *p < 0.05, **p < 0.01, ***p < 0.001 compared to control; ^#^*p* < 0.05 compared to different experimental group as marked by horizontal bar; only relevant significant values are highlighted. **a** Transient exposure to an acute inflammatory stimulus (24 h, followed by media change) of 10 ng/ml LPS (left and middle panel, red vertical bars) or 50 ng/ml IL4 (right panel, green vertical bar). INOS expression as a function of time after transient exposure was measured on the protein level by immunocytochemistry (*n* = 3, *H*(4) = 94.638, *p* < 0.001), NO release by Griess assay (μmol/l; *n* = 7, *H*(4) = 32.616, *p* < 0.001), and IGF1 release by ELISA (pg/ml, *n* = 5, *H*(4) = 10.128, *p* < 0.05). Data were normalized to control. **b** Permanent exposure to a chronic inflammatory stimulus, applied over the entire observation period of 96 h, of 10 ng/ml LPS (left and middle panel, red horizontal bars) or 50 ng/ml IL4 (right panel, green horizontal bar). INOS expression as a function of time after the beginning of permanent exposure was measured on the protein level by immunocytochemistry (*n* = 3, *H*(4) = 95.861, *p* < 0.001), NO release was measured by Griess assay (μmol/l; *n* = 7, *H*(4) = 44.542, *p* < 0.001); IGF1 release was measured by ELISA (pg/ml, *n* = 5, *F* (4, 27) = 259.4, *p* < 0.001, ω = 0.98). Data were normalized to control

To further explore the effects of timing and duration of stimulus exposure on microglia polarization, we subjected microglia to either a transient 24 h-course of LPS (red vertical bar) or IL4 (green vertical bar), respectively (Fig. 4a), or permanently treated them with the respective agent over the entire 96-h observation period (Fig. 4b, horizontal bars). Transient exposure to LPS for 24 h, followed by media change, led to an abrupt and extensive upregulation of iNOS protein (*p* < 0.001, Fig. 4a, left panel), as well as to release of NO (*p* < 0.001, Fig. 4a, middle panel). However, another 24 h later, iNOS expression and NO release had declined again. Interestingly, while iNOS expression even further declined during the course of observation, NO release recommenced again without further external stimuli (*p* < 0.001, Fig. 4a). In contrast, transient exposure to IL4 for 24 h induced microglia to continuously release increasing amounts of IGF1 over the entire observation period (*p* < 0.01, Fig. 4a, right panel), suggesting that effective M2 polarization only required a single pulsed stimulus. On the other hand, permanent exposure to LPS or IL4, respectively, led to long-term polarization towards M1 or M2 microglia, respectively, with continuously increasing levels of characteristic marker expression (*p* < 0.001, Fig. 4b).

### Microglia affect key functions of neural stem cells (NSCs)

To elucidate the effects of the microglia secretome on NSCs, we collected “conditioned” cell culture medium from differently polarized microglia. Only serum-free microglia medium was used on NSCs; microglia function was not impaired by their cultivation in serum-free medium for up to 24 h, as assessed by their expression of NO and pro-inflammatory cytokines. Conditioned medium was obtained from (i) M1 microglia that had been exposed to LPS, (ii) M2 microglia that had been exposed to IL4, (iii) “hybrid” microglia that had been exposed to LPS plus IL4, and (iv) “untreated” microglia that had not been subjected to an inflammatory stimulus as control. Next, NSCs were exposed to those four types of conditioned media (M-untreated, M1, M2, or M-hybrid), with another three groups of NSCs serving as control: (i) untreated NSCs in regular medium, (ii) NSCs directly exposed to LPS, and (iii) NSCs directly exposed to IL4. The latter control groups served to exclude direct effects of the inflammatory mediators on NSCs.

**Fig. 5:**
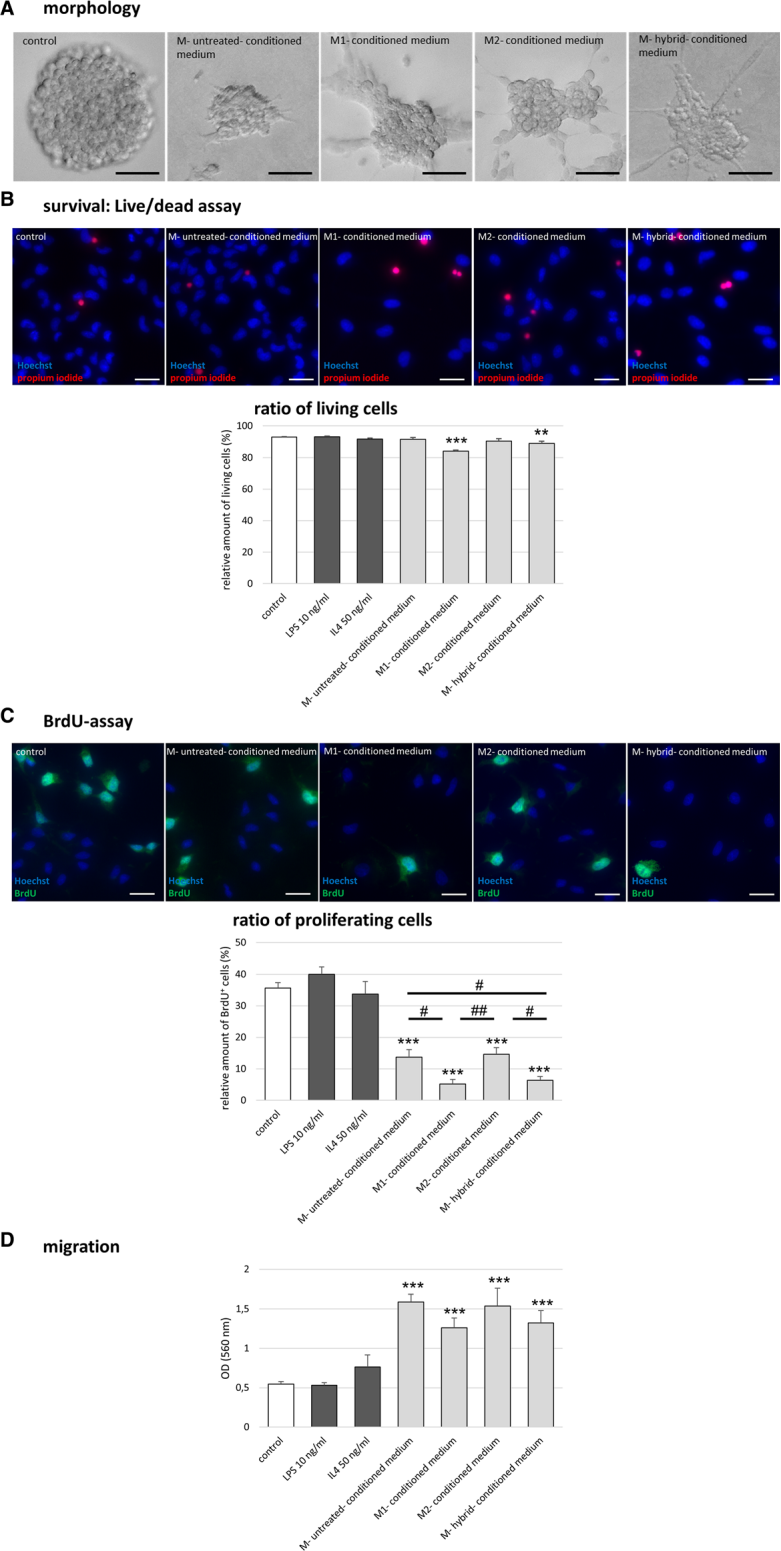
Microglia-conditioned media affect key neural stem cell (NSC) functions. **p < 0.01, ***p < 0.001 compared to control; ^#^*p* < 0.05, ^##^*p* < 0.01 compared to different experimental group as marked by horizontal bar; only relevant significant values are highlighted. **a** Representative images of neurospheres grown for 48 h in the presence of microglia-conditioned medium obtained from “M1” microglia exposed to LPS (10 ng/ml), “M2” microglia exposed to IL4 (50 ng/ml), “hybrid” microglia exposed to LPS plus IL4, or “untreated” microglia that had not been subjected to any inflammatory stimulus; scale bar = 50 μm. **b** Ratio of viable versus dead NSCs subjected to microglia-conditioned media as assessed by live/dead assay (*n* = 5, *H*(6) = 70.833, *p* < 0.001). Representative immunocytochemical images: all cells regardless of viability were stained by Hoechst (blue); dead cells were identified by propidium iodide incorporation (red); scale bar = 20 μm. **c** Proliferation rate of NSCs subjected to microglia-conditioned media as measured by BrdU incorporation (*n* = 7, *H*(6) = 218.951, *p* < 0.001). Representative immunocytochemical images: BrdU (green) identified proliferating cells, Hoechst as nuclear counterstain (blue); scale bar = 20 μm. **d** The migratory potential of NSCs subjected to microglia-conditioned media as assessed by Boyden chamber assay (*n* = 5, *H*(6) = 64.214, *p* < 0.001)

First, NSCs were grown as neurospheres in order to characterize their morphology (Fig. 5a). Upon stimulation with each of the four conditioned media for 48 h, neurospheres lost their spherical shape and displayed an irregular surface. In particular, neurospheres cultured in M1-conditioned medium adhered to the surface, reflecting partial spontaneous differentiation and a loss of stemness (Fig. 5a).

For all further experiments, and in order to further characterize key NSC functions, cells were grown as a monolayer culture of homogenous and undifferentiated NSCs. The effect of microglia-conditioned media on NSC survival was assessed via live/dead assay. Figure 5b demonstrates that both M1- as well as M-hybrid-conditioned medium led to a significant decrease in the percentage of viable NSCs (93% viable untreated NSC, 84% with M1-conditioned medium [*p* < 0.001], 89% with M-hybrid-conditioned medium [*p* < 0.01]), suggesting a negative impact of M1-microglia on NSC survival. The effect of microglia-conditioned media on NSC proliferation was assessed via BrdU incorporation. Average proliferation rate was 36% under control conditions (untreated NSCs exposed to BrdU for 6 h). Exposure to any microglia-conditioned medium reduced the proliferative activity of NSCs more than 50% (Fig. 5c). This decrease in NSC proliferation was most pronounced after exposure to M1-conditioned medium (95% reduction, *p* < 0.001) and M-hybrid-conditioned medium (94% reduction, *p* < 0.001; Fig. 5c). As a third key function of NSCs, their migratory potential was assessed by the Boyden chamber assay. Figure 5d demonstrates that exposure to any microglia-conditioned medium significantly increased the migratory activity of NSCs, suggesting a chemoattractant effect of the microglia secretome independent of microglia polarization (*p* < 0.001).

### Polarized microglia differentially affect NSC differentiation fate in vitro and in vivo

Upon mitogen withdrawal in vitro, NSCs are capable of differentiating into neurons, astrocytes, and oligodendrocytes. In order to detect the effects of microglia-conditioned media on the differentiation potential of NSCs, at the time of mitogen withdrawal, cells were exposed to the four different types of conditioned media (M-untreated, M1, M2, or M-hybrid) and compared to the three control groups (LPS alone, IL4 alone, or regular media).

**Fig. 6:**
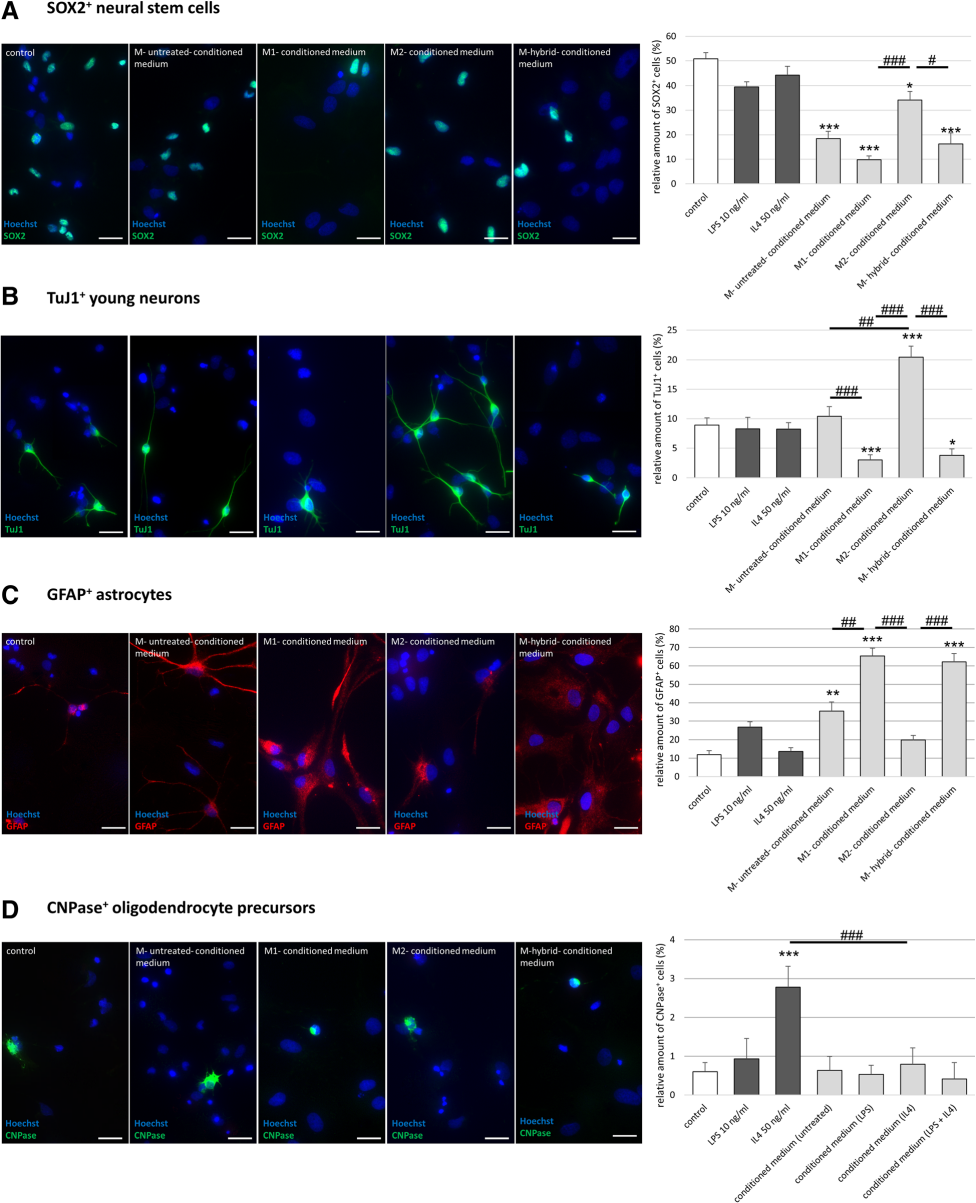
Polarized microglia differentially affect NSCs differentiation fate. *p < 0.05, **p < 0.01, ***p < 0.001 compared to control; ^#^*p* < 0.05, ^##^*p* < 0.01, ^###^*p* < 0.001 compared to different experimental group as marked by horizontal bar; only relevant significant values are highlighted. **a** Differentiation speed of NSCs subjected to microglia-conditioned media upon mitogen withdrawal as assessed by loss of “sex determining region Y-box 2” (SOX2) immunoreactivity (*n* = 6, *H*(6) = 123.685, *p* < 0.001); representative images stained for SOX2 (green), identifying undifferentiated NSCs, with Hoechst (blue) as nuclear counterstain; scale bar = 20 μm. **b** Neurogenic potential of NSCs subjected to microglia-conditioned media as assessed by “neuron specific beta-III tubulin 1” (TuJ1) immunocytochemistry (*n* = 6, *H*(6) = 80.356, *p* < 0.001); representative images stained for TuJ1 (green), identifying young neurons, with Hoechst (blue) as nuclear counterstain; scale bar = 20 μm. **c** Generation of astrocytes from NSCs subjected to microglia-conditioned media (*n* = 6, *H*(6) = 114.744, *p* < 0.001); representative images stained for “glial fibrillary acidic protein” (GFAP; red), identifying astrocytes, with Hoechst (blue) as nuclear counterstain; scale bar = 20 μm. **d** Generation of oligodendrocytes from NSCs subjected to microglia-conditioned media (*n* = 6, *H*(6) = 37.860, *p* < 0.001); representative images stained for “2′,3′-cyclic-nucleotide 3′-phosphodiesterase” (CNPase; green), identifying oligodendrocytes, with Hoechst (blue) as nuclear counterstain; scale bar = 20 μm

In order to determine the speed of NSC differentiation, cells were stained for SOX2 as a marker of (still) undifferentiated NSCs after 5 days of mitogen withdrawal (Fig. 6a). In the presence of any microglia-conditioned medium, this number of undifferentiated NSCs was significantly reduced compared to control, suggesting each microglia medium to accelerate the speed of NSC differentiation. However, this acceleration of differentiation was most pronounced in NSCs exposed to M1-conditioned medium (10% of NSCs remaining undifferentiated vs. 50% in control, *p* < 0.001, and much less profound after exposure to M2-conditioned medium (34% of NSCs remaining undifferentiated, *p* < 0.001 compared to M1-conditioned medium), suggesting that M1 microglia particularly accelerated NSC differentiation (Fig. 6a).

Next, we investigated the predominant fate of NSCs in the presence of microglia-conditioned media after 5 days of differentiation. M2-conditioned medium led to a significant increase in neurogenesis (20% TuJ1+ neurons compared to 10% in the control group, *p* < 0.001, while M1-conditioned medium decreased neurogenesis to 3% (*p* < 0.001 compared to control; Fig. 6b, left panel). Moreover, the dendrites of neurons generated from NSCs in the presence of M2-conditioned medium were longer than under control conditions (average of 82 μm compared to 49 μm in the control group, *p* < 0.05 (representative images Fig. 6b, and statistics in Additional file 3: Figure S3). On the other hand, M1-conditioned media promoted the generation of GFAP+ astrocytes from differentiating NSC (65% versus 12% under control conditions, *p* < 0.001, Fig. 6c). Of note, astrocytes generated from NSCs in the presence of M1- or M-hybrid-conditioned media appeared bigger and more mature in comparison to astrocytes generated under control conditions (Fig. 6c, representative images). We observed no effect of microglia-conditioned media on the generation of oligodendrocytes from NSCs; however, mere exposure to IL4 increased the percentage of oligodendrocytes generated from NSCs (*p* < 0.001, Fig. 6d), while neither LPS alone nor IL4 alone had any other effect on NSC differentiation (Figs. 6a–d).

**Fig. 7:**
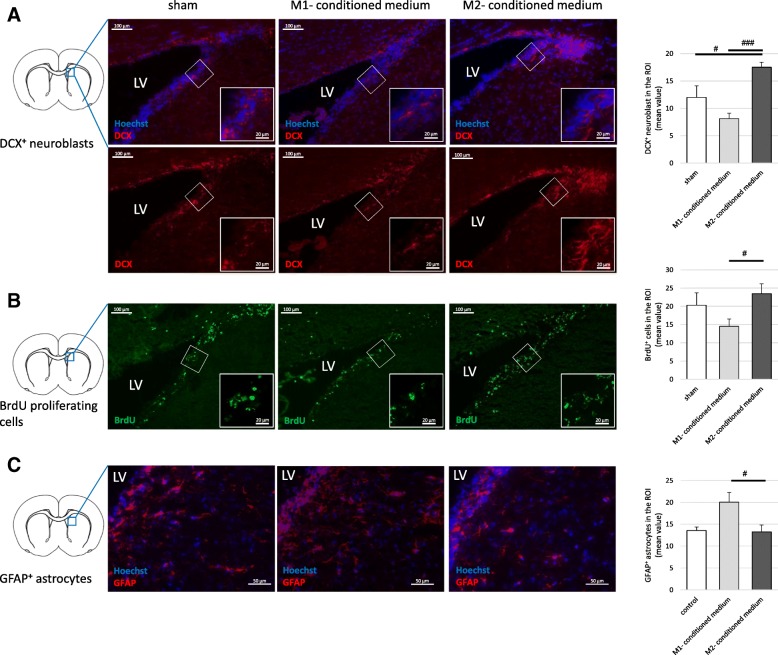
Effects of M1- and M2-conditioned microglia media on endogenous NSCs in vivo. ^*#*^*p* < 0.05, ^##^*p* < 0.01, ^###^*p* < 0.001 compared to different experimental group as marked by horizontal bar. Male adult Wistar rats received a single intracerebroventricular (i.c.v.) injection of M1-conditioned medium (*n* = 5), M2-conditioned medium (*n* = 5), or regular culture medium as control (*n* = 4). All animals received bromodeoxyuridine (BrdU) over 5 days and were sacrificed after 8 days for immunohistochemistry. **a** Effects of microglia-conditioned media on neurogenesis in the subventricular zone (SVZ) as assessed by staining for doublecortin (DCX)-positive neuroblasts (*F*(2, 19) = 14.215, *p* < 0.001, *ω* = 0.74). Representative images of DCX-immunohistochemistry (red) with Hoechst (blue) as nuclear counterstain; scale bar = 100 μm; in the magnified insert 20 μm. **b** Effects of microglia-conditioned media on proliferation in the SVZ as assessed by staining for BrdU (*F*(2, 19) = 2.443, *p* < 0.1, *ω* = 0.34). Representative images of BrdU immunohistochemistry (green); scale bar = 100 μm; in the magnified insert 20 μm. **c** Effects of microglia-conditioned media on the generation of astrocytes in the striatum directly adjacent to the SVZ (*H*(2) = 7.936, *p* < 0.05). Representative images of GFAP immunohistochemistry (red) with Hoechst (blue) as nuclear counterstain; scale bar = 50 μm

We aimed to reproduce these in vitro findings on the effects of microglia-conditioned media in vivo. Healthy adult rats received a single intracerebroventricular (i.c.v.) injection of M1- or M2-conditioned medium, respectively; animals injected with regular culture medium served as controls (labeled “sham”). Compared to sham-treated animals, the number of DCX± neuroblasts in the SVZ was increased in animals treated with M2-conditioned medium (*p* < 0.05, Fig. 7a). Furthermore, the numbers of DCX+ as well as BrdU± cells in the SVZ were significantly decreased in animals treated with M1-conditioned medium, in comparison with animals treated with M2-conditioned medium (*p* < 0.001 and *p* < 0.05, respectively, Figs. 7a, b). Simultaneously, exposure to M1-conditioned medium increased the number of GFAP+ astrocytes in the SVZ and in the striatum in vivo (*p* < 0.05 compared to M2-conditioned medium (Fig. 7c).

## Discussion

**Fig. 8:**
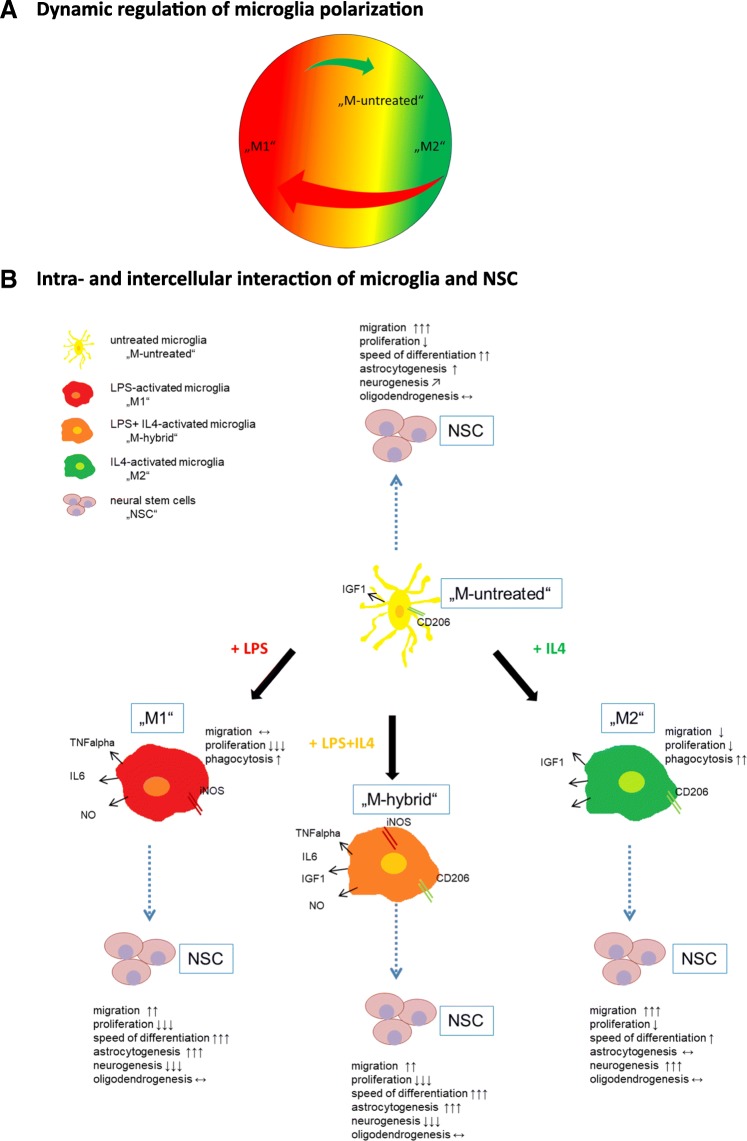
Overview of the experimental results and interpretation. **a** Schematic overview of the dynamic regulation of microglia polarization: primary untreated microglia (yellow) can be polarized to an M1 phenotype (red) or M2 phenotype (green) in vitro. Prior to exposure to inflammatory mediators, primary microglia in vitro are close to the M2 phenotype regarding characteristic expression patterns. Switching between polarization states using distinct inflammatory mediators such as LPS or IL4 is possible, however much more effective when transforming M2 microglia towards the M1 phenotype (red arrow) than vice versa (green arrow). **b** Untreated microglia (yellow) are polarized towards an M1 phenotype (red) following exposure to LPS, or to an M2 phenotype (green) in the presence of IL4. Simultaneous exposure to LPS plus IL4 results in a hybrid phenotype (orange) expressing both M1- and M2-characteristic markers. While microglia of all polarization states have similar effects on NSCs (pink) in regard to proliferation and migration, they characteristically differ in their effects on NSCs differentiation potential

Our study elaborates the complex conditions governing microglia polarization and the effects of differentially polarized microglia on key functions of NSCs. In summary, microglia were polarized towards an M1 phenotype following exposure to LPS, or to an M2 phenotype in the presence of IL4 (cf. Figs. 1 and 8b), while simultaneous exposure to LPS plus IL4 resulted in a hybrid phenotype expressing both M1- and M2-characteristic markers (cf. Figs. 3a and 8b). M2 microglia were less motile but displayed a higher phagocytic activity than M1 microglia (cf. Fig. 2). Switching between polarization states using defined inflammatory mediators was possible, however much more effective when transforming M2 microglia towards the M1 phenotype than vice versa (cf. Figs. 3b and 8a). To maintain M1 polarization, constant external stimulation with LPS was necessary, while effective M2 polarization only required a single pulsed stimulus of IL4 (cf. Fig. 4). Polarized microglia had distinct and differential effects on the differentiation potential of NSCs in vitro and in vivo, with M1 microglia promoting the generation of astrocytes, while M2 microglia rather supported neurogenesis (cf. Figs. 6, 7, and 8b). Regardless of their polarization, microglia inhibited NSC proliferation, increased NSC migration behavior, and accelerated NSC differentiation upon mitogen withdrawal (cf. Figs. 5, 6, and 8b).

The designation of the “classical” M1- and the “alternative” M2 polarization state originates from research on peripheral macrophages that polarize differently upon stimulatory signals from T helper cell type 1 (Th-1) and T helper cell type 2 (Th-2), respectively [34]. Since microglia as the brain’s resident immune cells share the peripheral macrophages’ capacity to polarize towards a cytotoxic (M1) or a phagocytotic (M2) phenotype [35, 36], the corresponding microglial activation subtypes were named M1 and M2 as well, in conformity with the macrophage nomenclature [37]. In recent years, the expression of cytokines, chemokines, and other molecules characteristic for each polarization phenotype has been species-specifically characterized both in vitro and in vivo [38]. Accordingly, the markers and releasing factors used in our study are widely accepted today and categorize our LPS-treated cells into a pro-inflammatory M1-treated cells and IL4-treated cells into an anti-inflammatory M2 phenotype [13]. However, macrophages and microglia differ in several regards. In vivo lineage tracing studies established that adult parenchymal microglia derive from primitive myeloid progenitors arising before embryonic day 8, identifying microglia as an ontogenetically distinct population in the mononuclear phagocyte system [39, 40]. Girard et al. highlighted that the function and effects of alternatively activated macrophages on surrounding neuronal tissue differ fundamentally from that of alternatively activated microglia [41]. Therefore, it is not valid to uncritically apply results from macrophage research to microglia. Several more recent studies have therefore specifically studied microglia polarization [15, 38, 42, 43]. In the healthy brain, microglia physiologically express a cluster of genes that allow them to sense and screen their surroundings for inflammatory cues, to promote neuronal survival, to contribute to activity-dependent synaptic remodeling, and to phagocytose damaged cells [44–46]. Franco and Fernandez-Suarez suggest that the “resting” microglia phenotype behaves similarly to M2 microglia—likely as an attenuated protective phenotype—which is in line with our observation of untreated microglia expressing low levels of M2 characteristic markers but display no characteristics of the M1 phenotype [15]. Of note, the current common dichotomous classification of M1 versus M2 represents only two major extreme types of activated microglia. Considering the complex inflammatory signaling cascades occurring in distinct temporo-spatial patterns after brain injury such as stroke, it has been criticized that the dichotomy of M1 versus M2 might be an improper simplification [19, 47, 48]. Addressing this matter, we here demonstrate that microglia are capable (i) to rapidly shift or abolish their polarization phenotype with changing or receding external stimuli and (ii) to adopt a hybrid phenotype exhibiting both M1 and M2 characteristics. In line with that, previous single-cell level studies on peripheral macrophages in vitro and in vivo describe simultaneously expressed high levels of signature polarization genes across different polarization states [49–51]. Another important aspect is the ability of microglia to “switch” from one polarization state to another. Our data show that—somewhat counter-intuitively—LPS as a powerful stimulus for M1 polarization has a stronger effect if microglia were pre-stimulated towards M2 with IL4. In line with our findings, Chhor et al. observed a sensitizing effect of IL4 on subsequent LPS treatment in microglia [42]. By contrast, Michelucci et al. suggest that prior exposure to the M2 stimulus IL10 inhibits M1 polarization of microglia [37]. Ghisletti et al. demonstrated that LPS regulates specific enhancers that determine the transcription not only of pro-inflammatory genes but also of inducible transcription factors in macrophages, leading to an alternation of the responsiveness to further environmental stimuli [52]. All of these studies support the evidence that microglia are affected by prior “experiences,” thus develop mechanisms of molecular memory [21, 42]. This is most relevant in vivo, since microglia accumulation is still detectable months and years after brain injury [1, 53, 54], presumably keeping their “memory” from the initial insult. In this context, preceding insults to the brain such as radiation, (mild) traumatic brain injury, or cerebral ischemia might shape a long-term “microglia memory” that in turn may later affect the susceptibility of the CNS to any “second hit,” potentially even including neurodegenerative disorders.

Overall, data suggest that a simple bi-directional model of microglia polarization does not suffice. We, therefore, favor a dynamical model that reflects dependence on tissue- and context-specific stimuli. For macrophages, Mosser and Edwards suggested a “colour wheel of macrophage activation”, with three primary populations of macrophages arranged in a circle, the spaces in between those primary phenotypes covered by macrophages with overlapping/hybrid characteristics [51]. Based on our data, we suggest an analogous “colour wheel of microglia activation” that reflects the dynamic regulation of microglia polarization (Fig. 8a).

After an ischemic brain lesion, activated microglia release soluble factors mobilizing endogenous NSCs from their niches to support regeneration [23, 25, 26]. Endogenous NSCs mediate their beneficial effects not only by neuronal replacement, but rather exhibit immunomodulatory, neuroprotective, and re-myelinating properties [18, 27]. Unfortunately, the regenerative capacity of NSCs after damage to the brain—such as stroke—is insufficient to achieve full functional recovery, which might be due to inappropriate modulation of NSCs by the surrounding inflammatory milieu. Thus, deciphering the interactions between the microglia secretome and NSCs aims at specifically addressing and enhancing the regenerative capacity of NSCs. We here demonstrate that M1 microglia impair both survival and proliferation of NSCs and block neurogenesis while promoting the generation of astrocytes in vitro. In line with these observations in vitro, we here demonstrate an increased number of GFAP-positive astrocytes in vivo, whereas DCX-positive neuroblasts and BrdU-positive proliferating cells in the SVZ were diminished after injection of the M1 secretome. Nevertheless, the situation in vivo is far more complex and was not entirely unraveled in the current study, since regulation of neuronal plasticity and repair in the brain strongly depend on a “microglial-astrocyte-neuron tripartite crosstalk” [55, 56]. Hence, besides the direct effects of M1 microglia-conditioned medium on NSCs in vivo described here, we might speculate about an additional activation of astrocytes that, in turn, can exert neurodegeneration and neurotoxic effects [57, 58]. Furthermore, L’Episcopo et al. recently demonstrated that the function of microglia-NSC-neuron cross-talk in the SVZ strongly varies with aging [55, 59]. Since we used fetal rat NSCs and microglia from newborn rats for our in vitro investigations, but adult animals for the in vivo experiments, effects might have been influenced by the age of the experimental animals. Nevertheless, in line with our findings, we recently discovered that a panel of pro-inflammatory cytokines (TNF-α, IL1β, and IL6) directly accelerates NSC differentiation and drives them towards an astrocytic cell fate in vitro [30]. After brain injury, astrocytes form a glial scar that—in the later stages of tissue remodeling after injury—blocks pivotal steps of repair and impedes axon regeneration [60–62]. Other studies point out that astrocytes also exert beneficial effects after injury: Faulkner et al. demonstrated that ablation of reactive astrocytes after spinal cord injury causes larger tissue damage, increased neuronal cell death, failure of blood-brain barrier repair, and worse functional outcome [63]. Shechter et al. report the scar formation itself to interact with infiltrating monocytes, driving them towards an anti-inflammatory phenotype which, in turn, resolves the scar-matrix again [64]. Overall, it is currently believed that not the astrocytic scar formation itself impairs recovery, but an improper timing of scar-forming and scar-resolution [64, 65]. The current study revealing strong effects of the M1 secretome on astrocytogenesis points out the high relevance of the cross-talk between microglia and NSCs in a neuroinflammatory milieu.

In comparison with the M1 secretome, the M2 secretome exhibited a comparatively weaker antiproliferative effect on NSCs in vitro and even a pro-proliferating effect in the cells of the SVZ in vivo while strongly increasing and accelerating the generation of new neurons in vitro and in vivo. In line with our observations, Yuan et al. recently demonstrated that primary mouse M2 microglia promote neurogenesis in NSCs in vitro [66]. On the other hand, Osman et al. reported no effect of M2 microglia derived from immortalized BV2 cells on the percentage of newly formed neurons from NSCs in vitro [67]. This discrepancy might be due to the different source of microglia used in the cited studies. Moreover, we did not observe an impact of any microglia-conditioned media on the generation of oligodendrocytes from NSCs, while other groups had described M2 microglia to promote oligodendrocytogenesis [66, 68, 69]. However, we found the characteristic M2 cytokine IL4 to indeed promote the generation of oligodendrocytes, when NSCs were directly exposed to it at high concentrations, suggesting a dose-dependent effect of IL4 on NSCs. To our knowledge, we here for the first time describe any direct effects of IL4 on NSCs. Regarding some remaining discrepancies between our data and a few previous studies, it must be noted that we used primary microglia derived from rat, while several previous studies dealt with mouse cells. Relevant species differences were recently described for microglia regarding cytokines as well as receptors expressed [38]. Moreover, for the first time, we here describe a hybrid phenotype of microglia that simultaneously exhibited both M1 and M2 characteristics. Interestingly, the effects of this hybrid phenotype on NSCs largely resembled the effects of M1 microglia, suggesting that the M1 secretome might be more potent than the M2 secretome to modulate NSCs.

## Conclusion

Overall, data support prospective therapeutic strategies to pharmacologically modify microglia polarization. Understanding the molecular and cellular mechanisms underlying both protective and detrimental immune processes should lead to novel strategies to harness protective responses while mitigating detrimental ones. Currently, several drugs are being explored in order to increase the neuroprotective functions of microglia or to shift the microglial phenotype towards a neuroprotective polarization [15]. However, microglia exhibit compensatory mechanisms and tend to quickly regain their original phenotype. Thus, the heterogeneity of microglial activation makes it difficult to predict the outcome from therapeutic immunomodulatory approaches [70]. The current study contributes to our understanding of the therapeutic regulation of microglia and their concomitant effects on NSCs and therefore represents an important advance towards novel regenerative therapies in neurology.

## Additional files

Additional file 1: **Figure S1.** Survival of primary microglia in the presence of inflammatory mediators. * p < 0.05, ** p < 0.01, *** p < 0.001 compared to different experimental group as marked by horizontal bar. A) Release of lactate dehydrogenase (LDH) was measured photometrically (LDH assay) as a surrogate for cell death after treatment of microglia with LPS (10 ng/ml), IL4 (50 ng/ml) or both (LPS plus IL4). Lysed cells served as control (*n* = 7, H(4) = 35.818, p < 0.001). B) Ratio of viable versus dead (propium iodide-positive) microglia subjected to LPS (10 ng/ml), IL4 (50 ng/ml) or both (LPS plus IL4) as assessed by Live/dead assay (*n* = 4, H(3) = 9207, p < 0.05). (TIF 618 kb)

Additional file 2: **Figure S2.** Simultaneous stimulation of primary microglia with LPS plus IL4. * p < 0.05, ** p < 0.01, *** p < 0.001 compared to different experimental group as marked by horizontal bar; # p < 0.05, ## p < 0.01, ### p < 0.001 between 2 groups (t-test); only relevant significant values are highlighted. Co-stimulation of microglia with LPS (1 or 10 ng/ml) and IL4 (50 ng/ml), and resulting expression of M1 markers: INOS was measured on the protein level by immunocytochemistry (*n* = 3; H(4) = 87.213, *p* < 0.001), release of TNF-α (n = 3, F(4, 30) = 7947, *p* < 0.001, ω = 0.665) and IL6 (*n* = 3; H(4) = 13.353, *p* < 0.01; t-test: t(4) = 6.064, *p* < 0.01, d = − 4.2) were measured by ELISA. (TIF 696 kb)

Additional file 3: **Figure S3.** Quantification of the dendritic length of TuJ1-positive neurons. * p < 0.05, ** p < 0.01, *** p < 0.001 compared to control; Quantification of the dendritic length of TuJ1-positive neurons generated from NSCs subjected to microglia-conditioned media upon mitogen withdrawal (*n* = 3, F (4, 89) = 8.416, *p* < 0.001, ω = 0.49). (TIF 363 kb)

## Abbreviations

CNS: Central nervous system
DMEM: Dulbecco’s modified Eagle medium
FCS: Fetal calf serum
FGF: Fibroblast growth factor
i.c.v: Introcerebroventricular
IL: Interleukin
iNOS: Inducible nitric oxide synthetase
LPS: Lipopolysaccharides
NO: Nitric oxide
NSC: Neural stem cell
OD: Optical density
PBS: Phosphate buffered saline
PFA: Paraformaldehyde
RT-qPCR: Real-time quantitative PCR
