# Supplementary figures and images for: The plasticity of primary microglia and their multifaceted effects on endogenous neural stem cells in vitro and in vivo
Source: J Neuroinflammation. 2018 Aug 13;15:226. doi: 10.1186/s12974-018-1261-y (PMC6090672; doi:10.1186/s12974-018-1261-y)

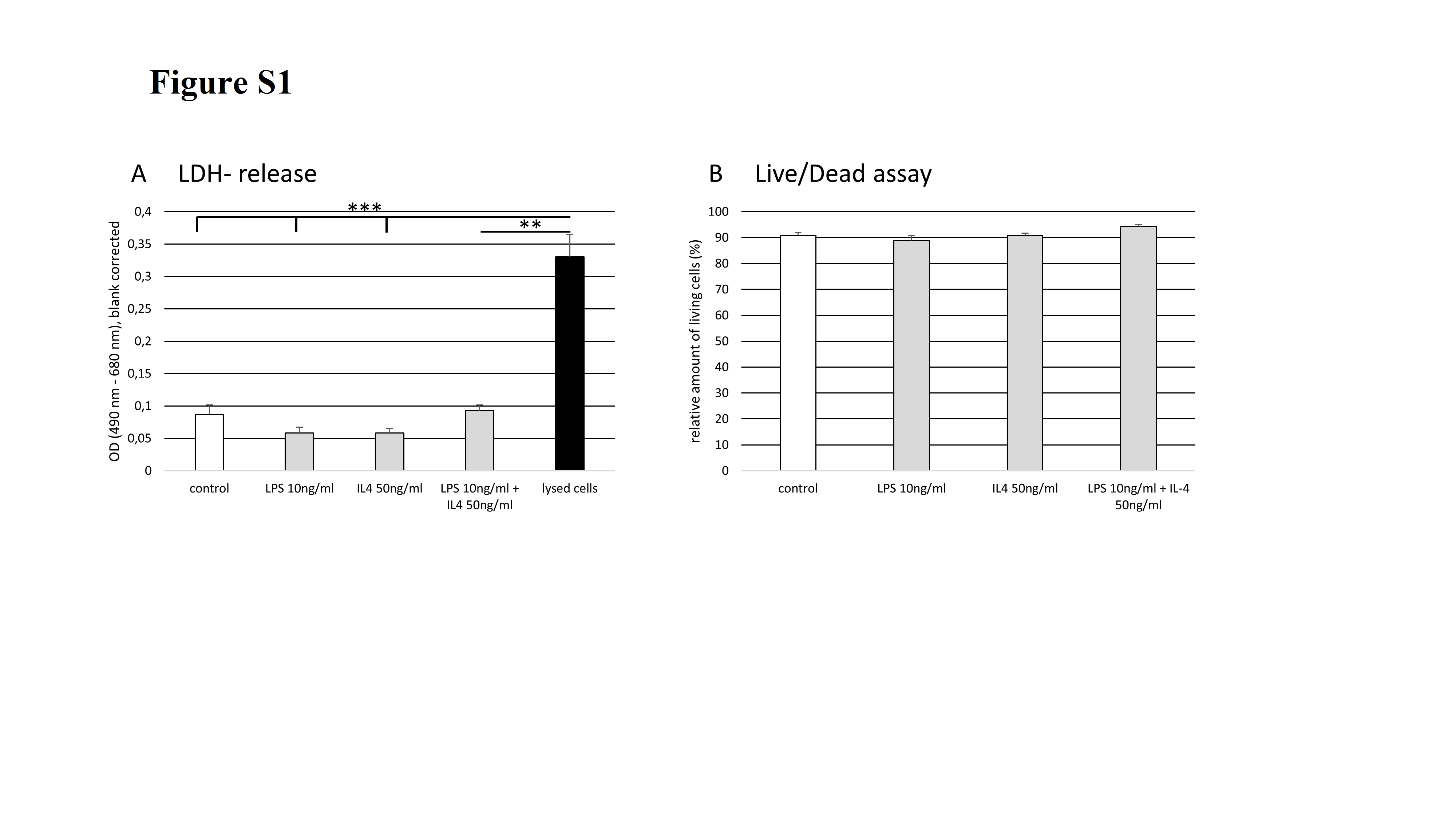

Supplement: Supplementary file 1 — Figure S1. Survival of primary microglia in the presence of inflammatory mediators. * p < 0.05, ** p < 0.01, *** p < 0.001 compared to different experimental group as marked by horizontal bar. A) Release of lactate dehydrogenase (LDH) was measured photometrically (LDH assay) as a surrogate for cell death after treatment of microglia with LPS (10 ng/ml), IL4 (50 ng/ml) or both (LPS plus IL4). Lysed cells served as control (n = 7, H(4) = 35.818, p < 0.001). B) Ratio of viable versus dead (propium iodide-positive) microglia subjected to LPS (10 ng/ml), IL4 (50 ng/ml) or both (LPS plus IL4) as assessed by Live/dead assay (n = 4, H(3) = 9207, p < 0.05). (TIF 618 kb) [file 12974_2018_1261_MOESM1_ESM.tif]

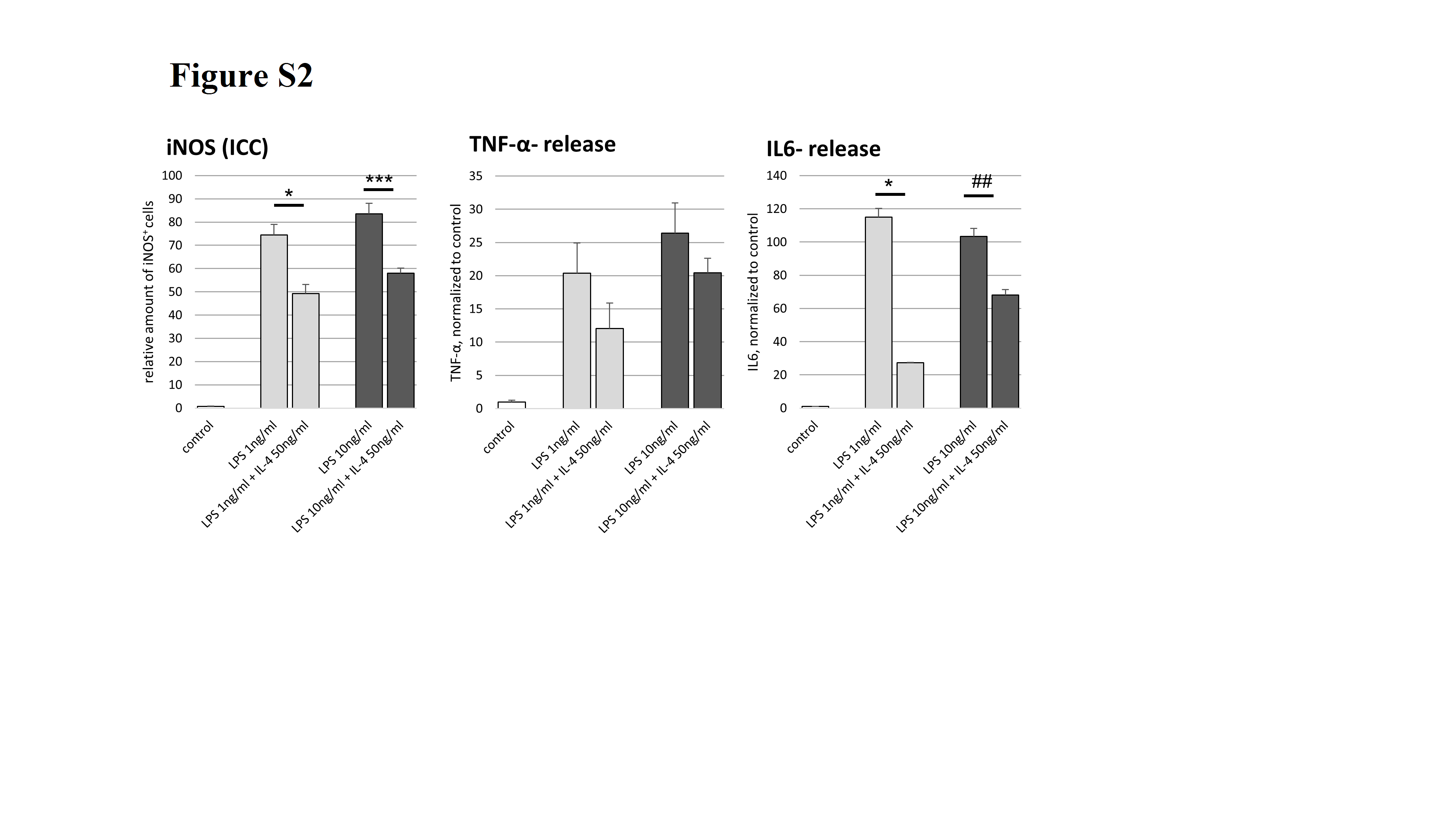

Supplement: Supplementary file 2 — Figure S2. Simultaneous stimulation of primary microglia with LPS plus IL4. * p < 0.05, ** p < 0.01, *** p < 0.001 compared to different experimental group as marked by horizontal bar; # p < 0.05, ## p < 0.01, ### p < 0.001 between 2 groups (t-test); only relevant significant values are highlighted. Co-stimulation of microglia with LPS (1 or 10 ng/ml) and IL4 (50 ng/ml), and resulting expression of M1 markers: INOS was measured on the protein level by immunocytochemistry (n = 3; H(4) = 87.213, p < 0.001), release of TNF-α (n = 3, F(4, 30) = 7947, p < 0.001, ω = 0.665) and IL6 (n = 3; H(4) = 13.353, p < 0.01; t-test: t(4) = 6.064, p < 0.01, d = − 4.2) were measured by ELISA. (TIF 696 kb) [file 12974_2018_1261_MOESM2_ESM.tif]

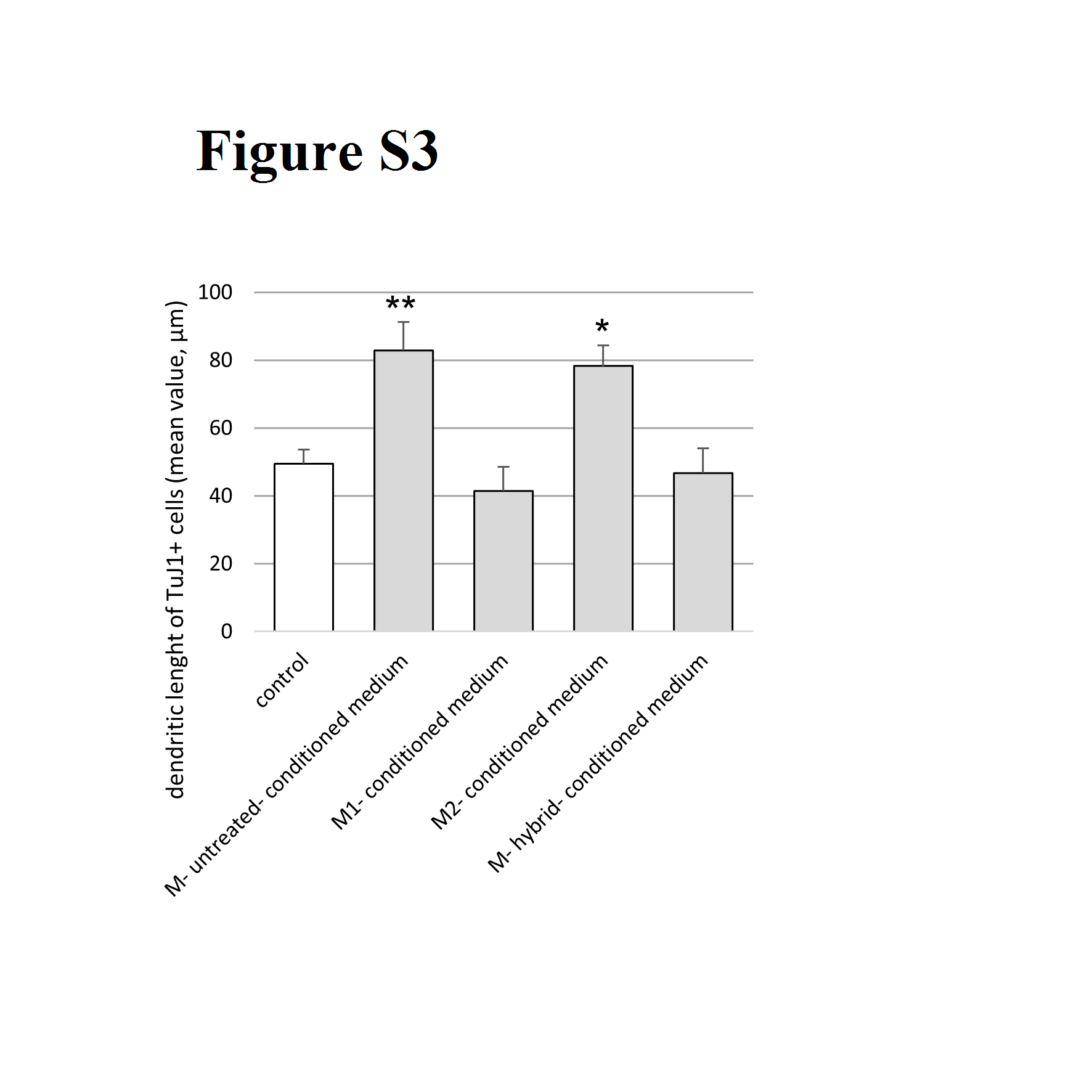

Supplement: Supplementary file 3 — Figure S3. Quantification of the dendritic length of TuJ1-positive neurons. * p < 0.05, ** p < 0.01, *** p < 0.001 compared to control; Quantification of the dendritic length of TuJ1-positive neurons generated from NSCs subjected to microglia-conditioned media upon mitogen withdrawal (n = 3, F (4, 89) = 8.416, p < 0.001, ω = 0.49). (TIF 363 kb) [file 12974_2018_1261_MOESM3_ESM.tif]
